# Entropic nonclassicality and quantum non-Gaussianity tests via beam splitting

**DOI:** 10.1038/s41598-019-54110-4

**Published:** 2019-11-28

**Authors:** Jiyong Park, Jaehak Lee, Hyunchul Nha

**Affiliations:** 1 School of Basic Sciences, Hanbat National University, Daejeon, 34158 Korea; 2 School of Computational Sciences, Korea Institute for Advanced Study, Seoul, 02455 Korea; 3 Department of Physics, Texas A&M University at Qatar, Education City, P.O.Box 23874 Doha, Qatar

**Keywords:** Theoretical physics, Quantum optics, Quantum information

## Abstract

We propose entropic nonclassicality criteria for quantum states of light that can be readily tested using homodyne detection with beam splitting operation. Our method draws on the fact that the entropy of quadrature distributions for a classical state is non-increasing under an arbitrary loss channel. We show that our test is strictly stronger than the variance-based squeezing condition and that it can also be extended to detect quantum non-Gaussianity in conjunction with phase randomization. Furthermore, we address how our criteria can be used to identify single-mode resource states to generate two-mode states demonstrating EPR paradox, i.e., quantum steering, via beam-splitter setting.

## Introduction

Nonclassicality is a concept of fundamenatal and practical importance in quantum optics and in quantum information using continuous variables (CVs). A single-mode light field is called nonclasssical if it cannot be represented as a probabilistic mixture of coherent states. It is a characterization of quantum light enabling optical phenomena unattainable in classical domain, which can also be used for many applications in quantum technology. The nonclassicality of a single-mode state generally provides resource for creating quantum entanglement via beam splitter setting (BS)^1–7^. Nonclassical squeezed states can also be employed for quantum metrology, e.g. phase estimation with better sensitivity than classical schemes^8–10^. In addition, nonclassical states are necessary for testing quantum foundations^11,12^. Recently, beyond the notion of nonclassicality, there has also been a growing interest in addressing quantum non-Gaussianity, which are regarded as an essential ingredient for quantum information processing. This is because Gaussian states and Gaussian operations have limited capabilities in some crucial tasks, e.g. quantum computation^13,14^, entanglement distillation^15–17^ and error correction^18^. A quantum state of light is called quantum non-Gaussian if it cannot be represented as a probabilistic mixture of Gaussian states. By its definition, the set of quantum non-Gaussian states belongs to the set of nonclassical states. The rigorous notion of quantum non-Gaussianity is introduced to detect higher-order optical processes that cannot be obtained by only Gaussian operations and their mixtures. Furthermore, the measures for quantum non-Gaussianity are developed by using a resource theoretical framework^19,20^.

We here propose experimentally feasible nonclassicality and quantum non-Gaussianity tests based on the entropy of quadrature distributions. Entropy, which quantifies the uncertainty about a random variable, is a key notion used in many branches of science including quantum foundation^21^, thermodynamics^22^, and information theory^23,24^. For continuous variable (CV) quantum information^25,26^, the entropy of quantum states has played a central role in establishing the capacities of Gaussian quantum channels^27–30^ and the entanglement of formation for Gaussian states^31,32^. It has also been employed for measuring non-Gaussianity^33–35^ and quantum non-Gaussianity^36^ of quantum states. Furthermore, the entropy of quadrature distributions has been used for assessing the performance of CV communication protocols, e.g., CV quantum key distribution^37,38^, CV quantum dense-coding^39,40^ and CV quantum communication in Gaussian regime^41–43^, and detecting quantum entanglement^44–46^ and quantum steering^47,48^.

The entropy of a quadrature distribtion can be readily obtained using a highly efficient tool of homodyne detection^49^. While the homodyne detection can also be used to test usual quadrature squeezing, which is a prototypical nonclassical property, we show that our criterion is strictly stronger than the squeezing criterion. That is, our method detects more nonclassical states than squeezing test. Our approach relies on the fact that the entropy of a quadrature distribution is non-increasing via loss channel for the case of a classical state. In other words, given a quantum state of light, if the output entropy turns out to be larger than the input entropy for a certain quadrature distribution, it is nonclassical. This test requires the measurement of quadrature distributions for the input and the output fields, respectively, which can be readily accomplished using homodyne detection with beam splitting operation. Our entropic test can be further extended to detect quantum non-Gaussianity in conjunction with phase randomization. Moreover, adopting two-quadrature version of our approach, we can identify a resource single-mode state that can demonstrate EPR paradox, i.e. quantum steering^50,51^, via a beam-splitter setting.

## Results

### Entropic nonclassicality criteria via beam splitting

For an input state $\rho$, the action of a loss channel can be described as

1 $$ {\mathcal L} [\rho ]={{\rm{Tr}}}_{B}[{\hat{B}}_{\eta }({\rho }_{A}\otimes |0\rangle \langle 0{|}_{B}){\hat{B}}_{\eta }^{\dagger }],$$

where ${\hat{B}}_{\eta }$ is a beam-splitting operation with transmittance $\eta$ between system *A* and environment *B*. Similarly, its complementary channel, which represents a reflected state instead of a transmitted state, is described as

2 $${\mathscr{C}}[\rho ]={{\rm{Tr}}}_{A}[{\hat{B}}_{\eta }({\rho }_{A}\otimes |0\rangle \langle 0{|}_{B}){\hat{B}}_{\eta }^{\dagger }].$$

We propose entropic criteria for nonclassicality as

3 $$\begin{array}{ll}{\bf{C}}{\bf{r}}{\bf{i}}{\bf{t}}{\bf{e}}{\bf{r}}{\bf{i}}{\bf{o}}{\bf{n}}\,{\bf{1}}\,{\boldsymbol{:}} &  {\mathcal F} [\rho ]\equiv {H}_{\rho }(Q)-{H}_{ {\mathcal L} [\rho ]}(Q) < 0,\end{array}$$

4 $$\begin{array}{ll}{\bf{C}}{\bf{r}}{\bf{i}}{\bf{t}}{\bf{e}}{\bf{r}}{\bf{i}}{\bf{o}}{\bf{n}}\,{\bf{2}}\,{\boldsymbol{:}} & {\mathscr{G}}[\rho ]\equiv {H}_{\rho }(Q)-{H}_{{\mathscr{C}}[\rho ]}(Q) < 0.\end{array}$$

**Figure 1:**
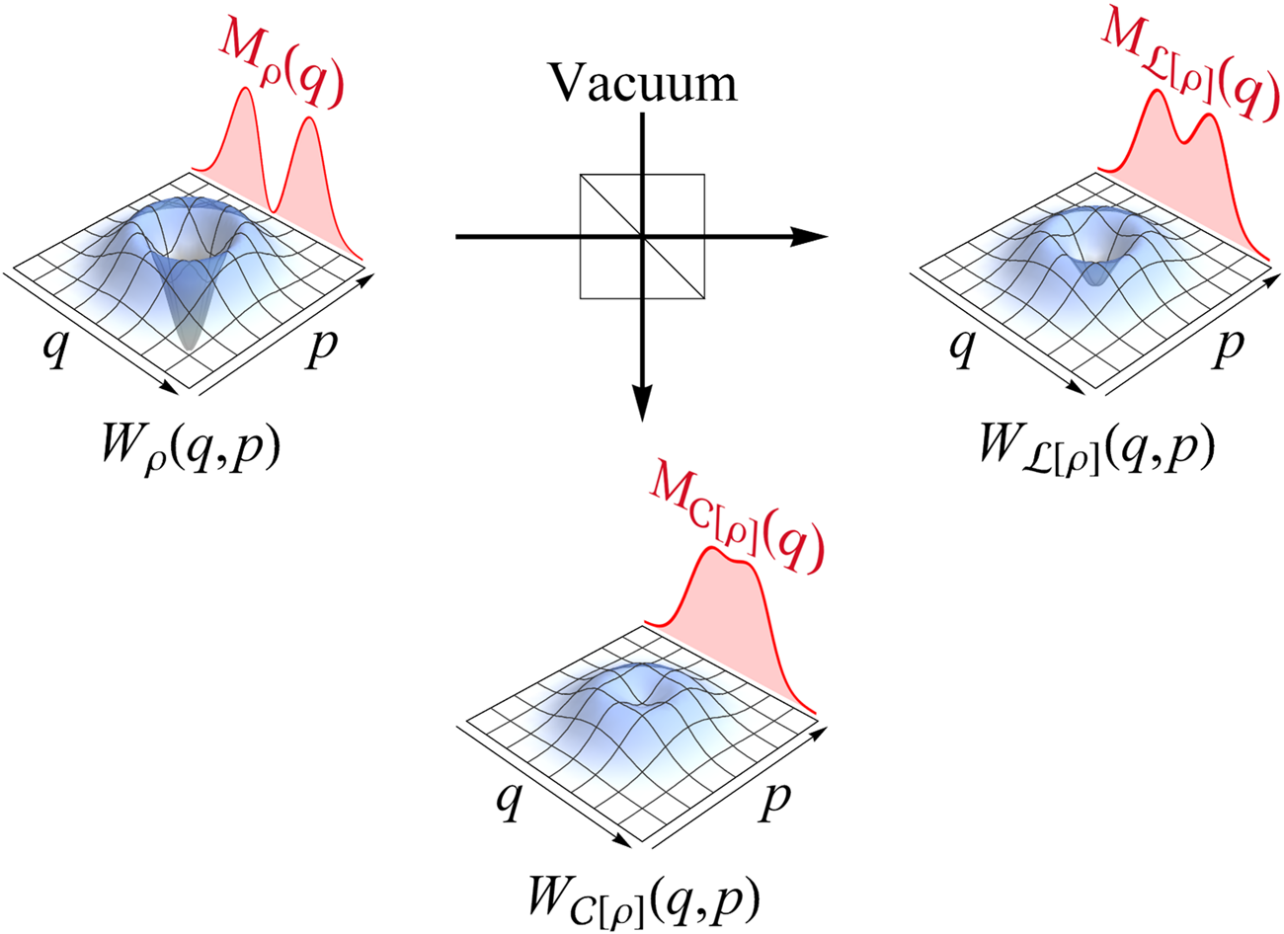
Scheme illustrating our nonclassicality criterion with an example of single-photon state $\rho =|1\rangle \langle 1|$. $W(q,p)$ represents each Wigner function while $M(q)=\int \,dpW(q,p)$ is its marginal distribution. ${\mathcal L} [\rho ]$ (${\mathscr{C}}[\rho ]$) is the transmitted (reflected) output via a beam splitter. If the entropy of the output distribution ${M}_{ {\mathcal L} [\rho ]}(q)$ or ${M}_{{\mathscr{C}}[\rho ]}(q)$ turns out to be larger than that of the input distribution ${M}_{\rho }(q)$, the original state $\rho$ is nonclassical.

Here the differential entropy ${H}_{\rho }(Q)$ is obtained by

5 $${H}_{\rho }(Q)=-\,{\int }_{-\infty }^{\infty }\,dq{M}_{\rho }(q)\,\log \,{M}_{\rho }(q),$$

in terms of quadrature distribution ${M}_{\rho }(q)=\int \,dp{W}_{\rho }(q,p)$ with ${W}_{\rho }(q,p)$ the Wigner function of $\rho$. That is, if the entropy of the output quadrature distribution is larger than that of the input distribution, the state is nonclassical (Fig. 1 for illustration). Its proof goes as follows.

**Proof:** When an input state $\rho$ is mixed with a vacuum state $|0\rangle$ at a beam splitter of transmissivity $t=\sqrt{\eta }$ and reflectivity $r=\sqrt{1-\eta }$, the output two-mode Wigner function is given by ${W}_{12}({q}_{1},{p}_{1},{q}_{2},{p}_{2})$ = ${W}_{\rho }(t{q}_{1}+r{q}_{2},t{p}_{1}+r{p}_{2}){W}_{|0\rangle \langle 0|}(\,-\,r{q}_{1}+t{q}_{2},-\,r{p}_{1}+t{p}_{2})$. The output quadrature distributions for transmitted and reflected fields are then given by

6 $$\begin{array}{rcl}{M}_{ {\mathcal L} [\rho ]}(q) & = & \int \,dpdq^{\prime} dp^{\prime} {W}_{12}(q,p,q^{\prime} ,p^{\prime} )\\  & = & {\int }_{-\infty }^{\infty }\,dq^{\prime} {M}_{\rho }(tq+rq^{\prime} ){M}_{|0\rangle \langle 0|}(\,-\,rq+tq^{\prime} ),\\ {M}_{{\mathscr{C}}[\rho ]}(q^{\prime} ) & = & \int \,dqdpdp^{\prime} {W}_{12}(q,p,q^{\prime} ,p^{\prime} )\\  & = & {\int }_{-\infty }^{\infty }\,dq{M}_{\rho }(tq+rq^{\prime} ){M}_{|0\rangle \langle 0|}(\,-\,rq+tq^{\prime} ),\end{array}$$

respectively. We first note that both of ${\mathcal F} [\rho ]$ and ${\mathscr{G}}[\rho ]$ defined in Eqs. (3) and (4) are concave with respect to states. Namely, ${\mathcal F} [{\sum }_{i}\,{p}_{i}{\rho }_{i}]\ge {\sum }_{i}\,{p}_{i} {\mathcal F} [{\rho }_{i}]$, and similarly for ${\mathscr{G}}$, of which proof is given in Methods.

With this concavity, for a classical state $\rho =\int \,{d}^{2}\alpha {P}_{\rho }(\alpha )|\alpha \rangle \langle \alpha |$ with a non-negative *P* function, we obtain

7 $$\begin{array}{rcl} {\mathcal F} [\int \,{d}^{2}\alpha {P}_{\rho }(\alpha )|\alpha \rangle \langle \alpha |] & \ge  & \int \,{d}^{2}\alpha {P}_{\rho }(\alpha ) {\mathcal F} [|\alpha \rangle \langle \alpha |]\\  & = & \int \,{d}^{2}\alpha {P}_{\rho }(\alpha )\{{H}_{|\alpha \rangle \langle \alpha |}(Q)-{H}_{ {\mathcal L} [|\alpha \rangle \langle \alpha |]}(Q)\}\\  & = & 0.\end{array}$$

In the above, we have used the facts that a coherent state transforms into another coherent state under a loss channel, i.e., ${\mathcal L} [|\alpha \rangle \langle \alpha |]=|t\alpha \rangle \langle t\alpha |$ with transmissivity *t*, and that the differential entropy of a quadrature distribution is identical for all coherent states, i.e., ${H}_{|\alpha \rangle \langle \alpha |}(Q)={H}_{|0\rangle \langle 0|}(Q)=\frac{1}{2}\,\log \,(\frac{\pi e}{2})$ for any *α*. The same argument applies to the entropy of the reflected state with ${\mathscr{C}}[|\alpha \rangle \langle \alpha |]=|r\alpha \rangle \langle r\alpha |$ and reflectivity *r*. That is, ${\mathscr{G}}[\int {d}^{2}\alpha {P}_{\rho }(\alpha )|\alpha \rangle \langle \alpha |]\ge 0$ for a classical state.

It is straightforward to see that the above analysis equally applies to an arbitrary quadrature amplitude ${\hat{q}}_{\theta }\equiv \hat{q}\,\cos \,\theta -\hat{p}\,\sin \,\theta$. Therefore, if the output entropy is larger than the input entropy for any quadrature distribution, the given state is confirmed to be nonclassical.

### Comparison with other nonclassicality tests

In this section, we compare our entropic nonclassicality criteria with the usual squeezing criterion. Before doing so, we also present an entropic form of squeezing condition, which constitutes another simple nonclassicality test^52^.

#### Entropic squeezing criterion

It is given by

8 $$\begin{array}{ll}{\bf{C}}{\bf{r}}{\bf{i}}{\bf{t}}{\bf{e}}{\bf{r}}{\bf{i}}{\bf{o}}{\bf{n}}\,{\bf{3}}\,{\boldsymbol{:}} & {H}_{\rho }(Q) < {H}_{|0\rangle \langle 0|}(Q),\end{array}$$

using the concavity of the differential entropy. That is, for a classical state,

9 $${H}_{{\rho }_{{\rm{cl}}}}(Q)\ge \int \,{d}^{2}\alpha {P}_{\rho }(\alpha ){H}_{|\alpha \rangle \langle \alpha |}(Q)={H}_{\mathrm{|0}\rangle \langle \mathrm{0|}}(Q\mathrm{).}$$

Equation (8) tells that the state is nonclassical if its quadrature entropy is less than that of a vacuum state, which is ${H}_{|0\rangle \langle 0|}(Q)=\frac{1}{2}\,\log (\frac{\pi e}{2})$.

*Remark*: We note that the entropic test in Eq. (8) can be considered as a subset of our previously proposed criteria, i.e., the so-called demarginalization map (DM) approach^53^. In DM method, nonclassicality is confirmed by showing the unphysicality of a fictitious Wigner function, e.g., constructed as ${W}_{DM}(q,p)={M}_{\rho }(q){M}_{|0\rangle \langle 0|}(p)$ where ${M}_{\rho }(q)$ is the quadrature distribution of a given state $\rho$ and ${M}_{|0\rangle \langle 0|}(p)=\sqrt{\frac{2}{\pi }}{e}^{-2{p}^{2}}$ that of a vacuum state. If a given state $\rho$ satisfies Eq. (8), we deduce

10 $$\begin{array}{ccc}{H}_{{\rho }_{{\rm{D}}{\rm{M}}}}(Q)+{H}_{{\rho }_{{\rm{D}}{\rm{M}}}}(P) & = & {H}_{\rho }(Q)+{H}_{|0\rangle \langle 0|}(P)\\  &  <  & \,{H}_{|0\rangle \langle 0|}(Q)+{H}_{|0\rangle \langle 0|}(P)\\  & = & \log (\frac{\pi e}{2}).\end{array}$$

Here *Q* and *P* represent the position and momentum quadratures, respectively, with their probability distributions ${M}_{\rho }(q)=\int \,dp{W}_{\rho }(q,p)$ and ${M}_{\rho }(p)=\int \,dq{W}_{\rho }(q,p)$ obtained from the Wigner function ${W}_{\rho }(q,p)$, and the differential entropies ${H}_{\rho }(Q)=-\,{\int }_{-\infty }^{\infty }\,dq{M}_{\rho }(q)\,\log \,{M}_{\rho }(q)$ and ${H}_{\rho }(P)=-\,{\int }_{-\infty }^{\infty }\,dp{M}_{\rho }(p)\,\log \,{M}_{\rho }(p)$. That is, ${\rho }_{{\rm{DM}}}$ violates the entropic uncertainty relation $H(Q)+H(P)\ge \,\log (\frac{\pi e}{2})$^21^, which means that ${\rho }_{{\rm{DM}}}$ is not a legitimate physical state confirming the nonclassicality of $\rho$.

#### Hierarchy of nonclassicality conditions

We here show that (i) the entropic squeezing condition in Eq. (8) is stronger than the usual variance-squeezing condition and that (ii) our main criteria in Eqs. (3) and (4) are stronger than the entropic squeezing condition in Eq. (8). Therefore, the hierarchy is given by {variance-squeezing} $\subset$ {entropic-squeezing} $\subset$ {entropy-nonclassicality via BS}.

**(i) {variance-squeezing}** $\subset$ **{entropic-squeezing}**

Suppose that a state, Gaussian or non-Gaussian, possesses a variance squeezing, i.e. ${V}_{\theta } < \frac{1}{4}$ for a certain quadrature ${\hat{q}}_{\theta }$. This condition can be expressed in terms of entropy as $h({V}_{\theta }) < h(\frac{1}{4})$, where $h(V)=\frac{1}{2}\,\log (2\pi eV)$ is the entropy of a Gaussian distribution with variance *V*. Now using the fact that a Gaussian distribution takes a maximal entropy under the same variance constraint, we deduce ${H}_{\rho }({Q}_{\theta })\le h({V}_{\theta }) < h(\frac{1}{4})={H}_{|0\rangle \langle 0|}({Q}_{\theta })$, which is nothing but the condition in Eq. (8).

On the other hand, there are states that have entropic squeezing but no variance-squeezing. Example is given in the next subsection. Therefore, the entropic squeezing condition is strictly stronger than the variance-squeezing condition.

**(ii) {entropic-squeezing}** $\subset$ **{entropy-nonclassicality via BS}**

Now suppose that a state satisfies ${H}_{\rho }(Q) < {H}_{|0\rangle \langle 0|}(Q)$. We here adopt an entropic inequality, i.e., $H(X{ \boxplus }_{\lambda }Y)\ge \lambda H(X)+(1-\lambda )H(Y)$^54,55^. $X{ \boxplus }_{\lambda }Y$ means the addition of two random variables *X* and *Y* with fractions $\sqrt{\lambda }$ and $\sqrt{1-\lambda }$, respectively. It is relevant to the action of a beamsplitter on the quadrature distributions. From the inequality, we deduce ${H}_{ {\mathcal L} [\rho ]}(Q)\ge \eta {H}_{\rho }(Q)+(1-\eta ){H}_{|0\rangle \langle 0|}(Q) > {H}_{\rho }(Q)$.

On the other hand, there are states that satisfy our entropic criteria in Eqs. (3) and (4) but not Eq. (8). Example is again given in the next subsection. Therefore our main entropic criteria are stictly stronger than the entropic squeezing, and also the usual variance-squeezing by (i).

#### Examples

We here illustrate the usefulness of our criteria by examples. We are particularly interested in non-Gaussian states without variance-squeezing, which can nevertheless be detected by our criteria in Eqs. (3), (4) and (8).

Our first example is a photon-added thermal state, i.e. ${\rho }_{{\rm{path}}}=\frac{{a}^{\dagger }{\rho }_{{\rm{th}}}a}{{\rm{Tr}}\{a{a}^{\dagger }{\rho }_{{\rm{th}}}\}}$ with ${\rho }_{{\rm{th}}}=\frac{1}{1+\bar{n}}\,{\sum }_{n}\,{(\frac{\bar{n}}{1+\bar{n}})}^{n}|n\rangle \langle n|$ a thermal state of mean number $\bar{n}$. Its quadrature distribution is given by

11 $$M(q)=\sqrt{\frac{2}{\pi }}\,\exp \,(\,-\,\frac{2{q}^{2}}{1+2\bar{n}})\frac{\bar{n}(1+2\bar{n})+4(1+\bar{n}){q}^{2}}{{(1+2\bar{n})}^{5/2}}.$$

**Figure 2:**
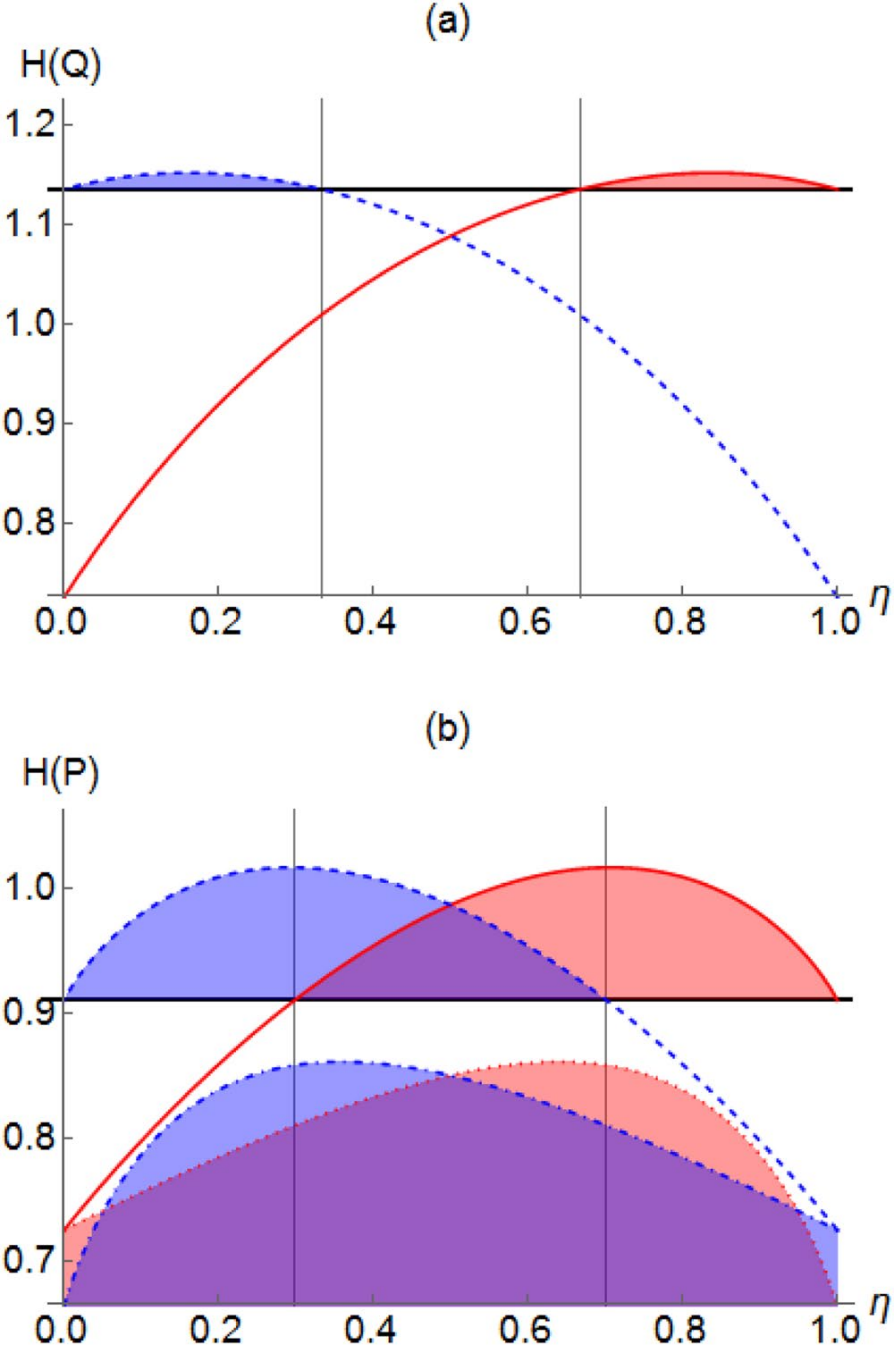
(**a**) Differential entropy *H*(*Q*) for a photon-added thermal state ${\rho }_{{\rm{path}}}$ with $\bar{n}=0.1$ against beam-splitting transmittance $\eta$. Black horizontal solid line represents *H*(*Q*) of the input ${\rho }_{{\rm{path}}}$ before beam splitting. On the other hand, red solid and blue dashed curves represent the output entropy of the transmitted field ${H}_{ {\mathcal L} [\rho ]}(Q)$ and that of the reflected field ${H}_{{\mathscr{C}}[\rho ]}(Q)$, respectively. Shaded area represents a successful detection of nonclassicality by Eqs. (3) and (4). (**b**) *H*(*P*) for an odd cat states $|\psi \rangle \sim |\gamma \rangle -|\,-\,\gamma \rangle$ with $\gamma =0.5$ (upper curves: red solid for ${H}_{ {\mathcal L} [\rho ]}(P)$ and blue dashed for ${H}_{{\mathscr{C}}[\rho ]}(P)$) and $\gamma =1$ (lower curves: red dotted for ${H}_{ {\mathcal L} [\rho ]}(P)$ and blue dot-dashed for ${H}_{{\mathscr{C}}[\rho ]}(P)$).

The quadrature distribution after beam splitting operation can be obtained using Eq. (6). We would like emphasize that the photon-added thermal state has no entropic squeezing, i.e., ${H}_{{\rho }_{{\rm{path}}}}(Q) > {H}_{\mathrm{|0}\rangle \langle \mathrm{0|}}(Q)$, for any $\bar{n}$. It is because of the rotational symmetry in phase space, i.e., ${H}_{{\rho }_{{\rm{path}}}}(Q)={H}_{{\rho }_{{\rm{path}}}}(P)$, and the entropic uncertainty relation, i.e., ${H}_{\rho }(Q)+{H}_{\rho }(P)\ge {H}_{|0\rangle \langle 0|}(Q)+{H}_{|0\rangle \langle 0|}(P)$, which must be satisfied by all quantum states. In Fig. 2(a), we show the differential entropy of ${\rho }_{{\rm{path}}}$ with $\bar{n}=0.1$ before and after a beam splitter of transmittance $\eta$. Black horizontal solid line represents the initial entropy ${H}_{\rho }(Q)\simeq 1.136$ whereas red solid and blue dashed curves represent the output entropy ${H}_{ {\mathcal L} [\rho ]}(Q)$ and ${H}_{{\mathscr{C}}[\rho ]}(Q)$, respectively. We see that our entropic criteria detect nonclassicality by using a beam splitter of transmittance in the range $\eta \gtrsim 0.667$ and $\eta \lesssim 0.333$, respectively, in view of Eqs. (3) and (4). We have numerically checked that the detectable range of $\eta$ decreases with $\bar{n}$ and that our criteria detect ${\rho }_{{\rm{path}}}$ for a thermal photon $0\le \bar{n}\lesssim 0.203$.

Our second example is an odd cat state $|\psi \rangle \sim |\gamma \rangle -|\,-\,\gamma \rangle$ whose quadrature distribution is given by

12 $$\begin{array}{rcl}M({q}_{\theta }) & = & \sqrt{\frac{2}{\pi }}\frac{\exp (\,-\,2{q}_{\theta }^{2}-2{\gamma }^{2}\,{\cos }^{2}\,\theta )}{1-\exp (\,-\,2{\gamma }^{2})}\\  &  & \times \,\{\,\cosh \,\mathrm{(4}\gamma {q}_{\theta }\,\cos \,\theta )-\,\cos \,\mathrm{(4}\gamma {q}_{\theta }\,\sin \,\theta \mathrm{)\}.}\end{array}$$

Here we look into the differential entropy of the momentum quadrature distribution, $\theta =\pi /2$, as it manifests entropic squeezing. That is, the odd cat state, even though it does not have variance squeezing at all, satisfies entropic squeezing condition in Eq. (8) for $\gamma \ge 0.891$. When the coherent amplitude is smaller $\gamma  < 0.891$, our main criteria in Eqs. (3) and (4) can detect its nonclassicality.

In Fig. 2(b), we illustrate the cases with $\gamma =0.5$ (upper curves) and $\gamma =1$ (lower curves). For $\gamma =0.5$, the input entropy ${H}_{\rho }(P)\simeq 0.911$ is less than the output entropy ${H}_{ {\mathcal L} [\rho ]}(P)$ (red solid) and ${H}_{{\mathscr{C}}[\rho ]}(P)$ (blue dashed) for $\eta \gtrsim 0.299$ and $\eta \lesssim 0.701$, respectively. On the other hand, if $\gamma \ge 0.891$, a beam splitter with any values of transmittance $\eta$ can be used to detect nonclassicality, as exemplified by the lower curves for $\gamma =1$.

### Entropic quantum non-Gaussianity criterion

A phase randomization can be useful in enhancing the performance of quantum information protocols^56,57^. Here we further extend our entropic approach to detect quantum non-Gaussianity by using beam-splitting in conjunction with phase randomization. A complete phase randomization can be described as

13 $$\rho \mapsto \frac{1}{2\pi }\,{\int }_{0}^{2\pi }\,d\theta {e}^{i\hat{n}\theta }\rho {e}^{-i\hat{n}\theta }.$$

The quadrature distribution of a phase randomized state ${\mathcal R} [\rho ]$ is written as

14 $${M}_{ {\mathcal R} [\rho ]}(q)=\frac{1}{2\pi }\,{\int }_{0}^{2\pi }\,d\theta {M}_{\rho }({q}_{\theta }\mathrm{).}$$

Similar to the previous analysis, we obtain due to concavity the relation

15 $${H}_{ {\mathcal R} [\rho ]}(Q)-{H}_{ {\mathcal L} \circ  {\mathcal R} [\rho ]}(Q)\ge \frac{1}{2\pi }\,{\int }_{0}^{2\pi }\,d\theta \{{H}_{\rho }({Q}_{\theta })-{H}_{ {\mathcal L} [\rho ]}({Q}_{\theta })\}.$$

If a given state is Gaussian, its entropy is solely deterimined by the covariance matrix $\Gamma$ whose elements are defined as ${\Gamma }_{ij}\equiv \frac{1}{2}\langle \Delta {\hat{x}}_{i}\Delta {\hat{x}}_{j}+\Delta {\hat{x}}_{j}\Delta {\hat{x}}_{i}\rangle$ with ${\hat{x}}_{1,2}=\hat{q},\hat{p}$. With the notation $$\Gamma =(\begin{array}{cc}{\Gamma }_{11} & {\Gamma }_{12}\\ {\Gamma }_{21} & {\Gamma }_{22}\end{array})$$, ${H}_{\rho }({Q}_{\theta })=\frac{1}{2}\,\log (2\pi e{V}_{\theta })$ with ${V}_{\theta }={\Gamma }_{11}\,{\cos }^{2}\,\theta +{\Gamma }_{22}\,{\sin }^{2}\,\theta$ for a quadrature amplitude ${\hat{q}}_{\theta }\equiv \hat{q}\,\cos \,\theta -\hat{p}\,\sin \,\theta$. Using $\log \,x\ge 1-\frac{1}{x}$, we now have

16 $$\begin{array}{rcl}\frac{1}{2\pi }\,{\int }_{0}^{2\pi }\,d\theta \{{H}_{\rho }({Q}_{\theta })-{H}_{ {\mathcal L} [\rho ]}({Q}_{\theta })\} & = & \frac{1}{\pi }\,{\int }_{0}^{\pi }\,d\theta \{{H}_{\rho }({Q}_{\theta })-{H}_{ {\mathcal L} [\rho ]}({Q}_{\theta })\}\\  & = & \frac{1}{\pi }\,{\int }_{0}^{\pi }\,d\theta \frac{1}{2}\,\log \,\frac{{V}_{\theta }}{\eta {V}_{\theta }+\frac{1-\eta }{4}}\\  & \ge  & \frac{1}{2\pi }\,{\int }_{0}^{\pi }\,d\theta (1-\eta -\frac{1-\eta }{4{V}_{\theta }})\\  & = & \frac{1-\eta }{2}-\frac{1-\eta }{8\pi }\,{\int }_{0}^{\pi }\,\frac{d\theta }{{\Gamma }_{11}\,{\cos }^{2}\,\theta +{\Gamma }_{22}\,{\sin }^{2}\,\theta }\\  & = & \frac{1-\eta }{2}(1-\frac{1}{4\sqrt{{\Gamma }_{11}{\Gamma }_{22}}})\ge 0.\end{array}$$

Note that we have used the uncertainty principle for position and momentum ${\Gamma }_{11}{\Gamma }_{22}\ge \frac{1}{16}$. In addition, the integration of $\frac{1}{{a}^{2}\,{\cos }^{2}\,\theta +{b}^{2}\,{\sin }^{2}\,\theta }$ can be done by introducing $u=b\,\tan \,\theta$ and using $\int \,\frac{du}{{a}^{2}+{u}^{2}}=\frac{1}{a}\,\arctan \,\frac{u}{a}$.

**Figure 3:**
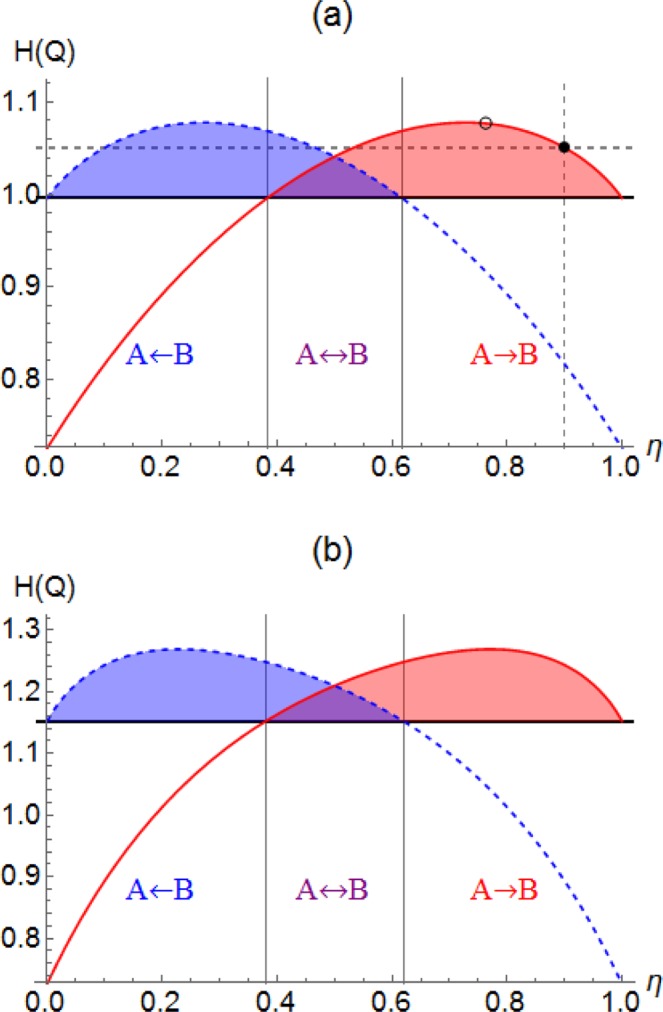
Differential entropy of (**a**) single-photon $\mathrm{|1}\rangle$ and (**b**) two-photon $\mathrm{|2}\rangle$ states under loss channel ${\mathcal L}$ (red solid) and its complementary channel ${\mathscr{C}}$ (blue dashed) with respect to transmittance $\eta$ of beam splitter. Black solid lines represent the entropy of each state before beam splitter.

The result in Eq. (16) indicates that the differential entropy of a phase-randomized Gaussian state always decreases after a loss channel. Using Jensen’s inequality again, we readily see that the same argument applies to a statistical mixture of phase-randomized Gaussian states. Therefore, if a quantum state $\rho$ satisfies

17 $$\begin{array}{ll}{\bf{C}}{\bf{r}}{\bf{i}}{\bf{t}}{\bf{e}}{\bf{r}}{\bf{i}}{\bf{o}}{\bf{n}}\,4{\boldsymbol{:}} & {H}_{ {\mathcal R} [\rho ]}(Q) < {H}_{ {\mathcal L} \circ  {\mathcal R} [\rho ]}(Q),\end{array}$$

the state is quantum non-Gaussian meaning that it cannot correspond to a probabilistic mixture of Gaussian states. Our criterion is particularly useful to detect quantum non-Gaussianity of a rotationally symmetric state in phase space, e.g. Fock states (Fig. 3).

### Resource for quantum steerability

Now we extend our approach further to involve entropies for two orthogonal quadrature amplitudes. By doing so, we can identify a single-mode resource state that can be used to demonstrate EPR paradox, i.e. quantum steering^50^, via a beam-splitter setting.

If a quantum state $\rho$ satisfies

18 $$\begin{array}{ll}{\bf{C}}{\bf{r}}{\bf{i}}{\bf{t}}{\bf{e}}{\bf{r}}{\bf{i}}{\bf{o}}{\bf{n}}\,5{\boldsymbol{:}} & {H}_{\rho }(Q)+{H}_{\rho }(P) < {H}_{ {\mathcal L} [\rho ]}(Q)+{H}_{ {\mathcal L} [\rho ]}(P),\end{array}$$

a two-mode quantum steerable state is produced by mixing $\rho$ with vacuum at a beam-splitter.

This can be seen by considering the entropic steering criterion proposed in ref.  ^47^ as

19 $${H}_{{\rho }_{AB}}({Q}_{B}|{Q}_{A})+{H}_{{\rho }_{AB}}({P}_{B}|{P}_{A}) < {H}_{|0\rangle \langle 0|}(Q)+{H}_{|0\rangle \langle 0|}(P)=\,\log (\frac{\pi e}{2}),$$

where conditional entropy ${H}_{{\rho }_{AB}}({Q}_{B}|{Q}_{A})$ is obtained by

20 $${H}_{{\rho }_{AB}}({Q}_{B}|{Q}_{A})=-\,\int \,d{q}_{B}\,\int \,d{q}_{A}{M}_{{\rho }_{AB}}({q}_{A},{q}_{B})\,\log \,\frac{{M}_{{\rho }_{AB}}({q}_{A},{q}_{B})}{{M}_{{\rho }_{A}}({q}_{A})}.$$

If Eq. (19) is satisfied, it demonstrates A to B steering^47^; All quantum states must satisfy the entropic uncertainty relation ${H}_{\rho }(Q)+{H}_{\rho }(P)\ge \,\log (\frac{\pi e}{2})$^21^. The condition in Eq. (19) means that the entropy of system B conditioned on the measurement outcome of system A beats this standard uncertainty relation due to quantum correlation, which evidences quantum steering^47^. On the other hand, one can also investigate B to A steering by interchanging indices $A\leftrightarrow B$ in Eq. (19).

Joint quadrature distribution of a quantum state ${\hat{U}}_{{\rm{BS}}}(\rho \otimes \mathrm{|0}\rangle \langle \mathrm{0|)}{\hat{U}}_{{\rm{BS}}}^{\dagger }$ is given by

21 $${M}_{{\rho }_{AB}}({q}_{A},{q}_{B})={M}_{\rho }(t{q}_{A}+r{q}_{B}){M}_{|0\rangle \langle 0|}(\,-\,r{q}_{A}+t{q}_{B}),$$

which gives

22 $${H}_{{\rho }_{AB}}({Q}_{B}|{Q}_{A})={H}_{\rho }(Q)+{H}_{|0\rangle \langle 0|}(Q)-{H}_{ {\mathcal L} [\rho ]}(Q).$$

Therefore, if the condition in Eq. (18) is satisfied, the two-mode state after the beam splitter satisfies the entropic steering condition in Eq. (19). As a remark, our criteria in Eqs. (17) and (18) are of course equally established using the complementary channel ${\mathscr{C}}[\rho ]$ representing the reflected field.

#### Example

Here we illustrate the cases of Fock states $\mathrm{|1}\rangle$ and $\mathrm{|2}\rangle$ whose quantum non-Gaussianity and usefulness for quantum steering can be demonstrated by our criteria in Eqs. (17) and (18). These states are rotationally symmetric in phase space, which leads to identical probability distributions for all quadrature distributions. Therefore, all of our proposed criteria are satisfied in the same parameter regions. That is, the shaded regions in Fig. 3(a,b) represent the successful detection of nonclassicality, quantum non-Gaussianity, and usefulness for quantum steering simultaneously.

In particular, quantum non-Gaussianity of states $\rho =|1\rangle \langle 1|$ and $\rho =|2\rangle \langle 2|$ can be confirmed by Eq. (17) for $0.383\lesssim \eta \le 1$ and $0.38\lesssim \eta \le 1$, respectively. One remark is in order. Beam-splitting operation is multiplicative, i.e. a BS with ${\eta }_{1}$ followed by another BS with ${\eta }_{2}$ correponds to a BS with $\eta ={\eta }_{1}{\eta }_{2}$. Examining carefully the curves in Fig. 3, we see that not only a pure Fock state but also a noisy Fock state under loss can be detected by our analysis. For instance, let a single-photon state undergo a loss channel with ${\eta }_{1}=0.9$ (filled circle in Fig. 3). The nonclassicality of this noisy output state can be detected by injecting it at a beam splitter of transmittance, e.g., ${\eta }_{2}=0.85$ (hollow circle in Fig. 3). This is because the quadrature entropy increases with decreasing $\eta$ from 1 to 0.728 in red curve of Fig. 3(a). The same argument can also be given for two-photon state, for which the entropy increases with decreasing $\eta$ from 1 to 0.771 in red curve of Fig. 3(b).

For quantum steering, we identify the regions for one-way steering and two-way steering, respectively. Each colored region, red or blue, represents the A (transmitted field) to B (reflected field) steering or vice versa. The overlap region in purple represents the steering in both of the ways.

#### Comparison with other QNG criteria

It may be interesting to compare our QNG criterion with other existing criteria particularly in refs. ^58–60^. Unlike the case of the nonclassicality criteria, we do not have a hierarchical relation among these existing QNG criteria and ours. That is, one criterion does not include another as a subset, but different criteria can be complementary to one another. For instance, while the criterion in ref. ^60^ is useful to detect QNG for a finite superposition of Gaussian states, e.g. generalized cat-states as discussed in ref. ^60^, it is not suitable to address QNG of Fock states. On the other hand, the other criteria in refs. ^58,59^ and our criterion successfully detect noisy Fock states to some extent.

**Figure 4:**
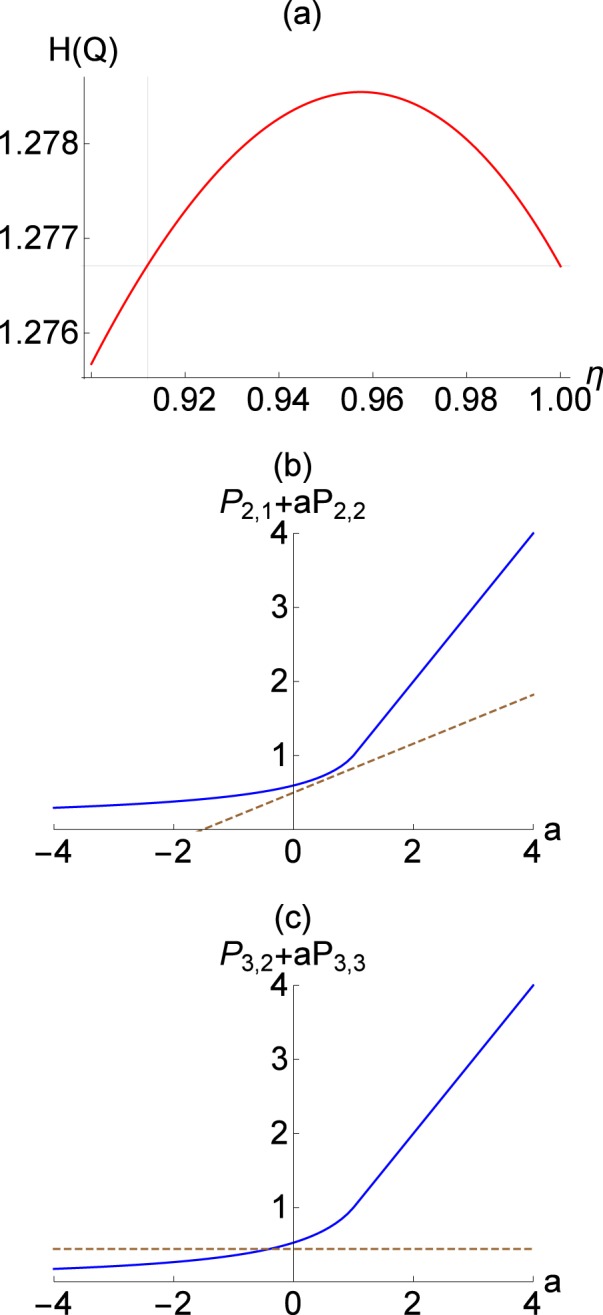
QNG detection of the state $\rho =0.17|0\rangle \langle 0|+0.17|1\rangle \langle 1|+0.66|2\rangle \langle 2|$ via (**a**) our criterion in Eq. (17) using a beam splitter of transmittance $\eta$ and (**b**,**c**) the criterion in ref. ^59^. In (**b**,**c**), QNG is detected if the value of ${P}_{2,1}+a{P}_{2,2}$ in (**b**), or ${P}_{3,2}+a{P}_{3,3}$ in (**c**), for the state $\rho$ (brown dashed lines) is above the Gaussian bound (blue solid lines) for a certain *a*. See main text for details.

We further compare our criterion and the one in ref. ^59^ by showing how Fock-diagonal states can be detected via each method. Reference^59^ introduced a QNG criterion in the form of ${P}_{n,k}+a{P}_{n,k+1}$, where ${P}_{n,k}$ represents a probability of firing *k*-detectors out of total *n*-detectors while *a* is a free parameter to optimize criterion. For each *a*, there exists a maximum value of ${P}_{n,k}+a{P}_{n,k+1}$ achieved by a whole class of Gaussian mixture states so that the value above this bound becomes the signature of QNG^59^. In Fig. 4, we show the results for the state $\rho =0.17|0\rangle \langle 0|+0.17|1\rangle \langle 1|+0.66|2\rangle \langle 2|$. As shown in Fig. 4(a), our criterion detect its QNG via the beam splitting of transmittance $\eta  > 0.912$. On the other hand, the criterion in ref. ^59^ does not detect QNG when the number of detectors is limited to two. That is, the value of ${P}_{2,1}+a{P}_{2,2}$ for the state $\rho$ (brown dashed line) does not go above the Gaussian bound (blue solid line) for any *a*. On the other hand, it can be made successful by increasing the number of detectors to three, i.e. ${P}_{3,2}+a{P}_{3,3}$ for the state $\rho$ (brown dashed line) beats the Gaussian bound (blue solid line) for a certain range of *a*.

The above example illustrates that the QNG criterion in ref. ^59^ requires an increasingly large number of detectors for higher Fock states, which may become less efficient with nonideal detector efficiency. However, ref. ^59^ also proposed a novel concept of QNG, i.e. genuine *n*-photon QNG, which certainly deserves a separate discussion elsewhere. In contrast, our criterion always uses the same experimental setup, i.e. homodyne detection known to be highly efficient, regardless of input states. From a fundamental point of view, it is also noteworthy that the criteria in refs. ^58,59^ consider the particle nature measuring photon-number distributions, whereas the one in ref. ^60^ and our criterion consider the wave nature measuring quadrature-amplitude distributions.

### Experimental feasibility

To test our entropic criterion, one just needs to measure a probability distribution of a quadrature amplitude ${M}_{\rho }({q}_{\phi })=\int \,dp{W}_{\rho }(q\,\cos \,\phi +p\,\sin \,\phi ,-\,q\,\sin \,\phi +p\,\cos \,\phi )$ before and after beam splitter. This probability distribution immediately gives the entropy ${H}_{\rho }({Q}_{\phi })=-\,{\int }_{-\infty }^{\infty }\,dq{M}_{\rho }({q}_{\phi })\,\log \,{M}_{\rho }({q}_{\phi })$. In quantum optics laboratory, homodyne detection is a well established, highly efficient, scheme to measure quadrature amplitudes constituting an integral part of state tomography^49^, thus our criterion is readily testable. On the other hand, there is one practical issue to consider for experimental feasibility. In a realistic homodyne detection, the measurement cannot discern the values of ${q}_{\varphi }$ within an interval of size *σ*, where *σ* represents the size of data binning. It leads to a coarse-grained probability distribution, instead of smooth continuous distribution^49,61^, as

23 $$\tilde{M}({q}_{\varphi })=\mathop{\sum }\limits_{n=-\infty }^{\infty }\,{M}_{\sigma }[n,\varphi ]\,{\rm{rect}}(\frac{q}{\sigma }-n),$$

with a step function ${\rm{rect}}(x)$

24 $${\rm{rect}}(x)=\{\begin{array}{ll}1 & {\rm{for}}\,|x|\le 1/2,\\ 0 & {\rm{for}}\,|x| > 1/2.\end{array}$$

Here ${M}_{\sigma }[n,\varphi ]$ is the coarse-grained probability averaged in the interval of $q\in [(n-\frac{1}{2})\sigma ,(n+\frac{1}{2})\sigma ]$ as

25 $${M}_{\sigma }[n,\varphi ]\equiv \frac{1}{\sigma }\,{\int }_{(n-\frac{1}{2})\sigma }^{(n+\frac{1}{2})\sigma }\,dxM({q}_{\varphi }).$$

This step-wise discontinuous distribution typically adds more entropy owing to information loss. To rigously address our criterion with coarse-graining *σ*, we come up with the entropic bound of nonclassicality due to *σ* in Methods. There we find the modified criterion of nonclassicality as ${H}_{\rho }(Q)+{B}_{\sigma } < {H}_{ {\mathcal L} [\rho ]}(Q)$, where ${B}_{\sigma } > 0$ can be readily obtained numerically for each *σ*. That is, the condition of having a higher entropy after the loss channel still works with a nonzero adjustment *B*_*σ*_.

**Figure 5:**
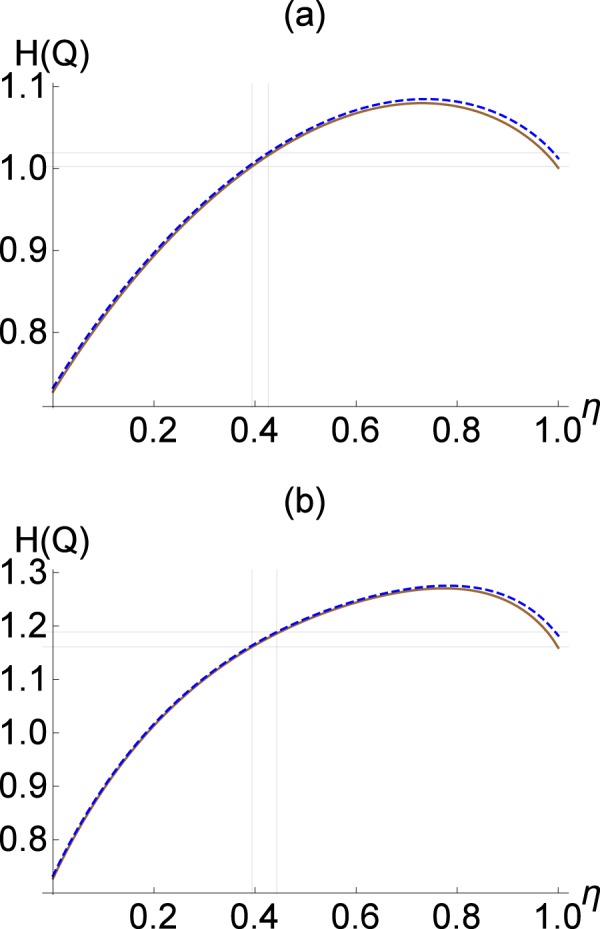
Differential entropy of (**a**) single-photon $\mathrm{|1}\rangle$ and (**b**) two-photon $\mathrm{|2}\rangle$ states under loss channel ${\mathcal L}$ with transmittance $\eta$ of beam splitter. The entropies here correspond to those resulting from a coarse-grained probability distribution with a binning size $\sigma =0.1$ (brown solid) and 0.2 (blue dashed) for each state.

In Fig. 5, we show the results of our nonclassicality test with coarse-graining. We first numerically obtain the bound *B*_*σ*_ = 0.0016639 and 0.0066226 for *σ* = 0.1 and *σ* = 0.2, respectively. As shown in the plots, our approach is still successful in detecting Fock states with finite binning. For a single photon state, we can detect it by using $0.394 < \eta  < 1$ and $0.426 < \eta  < 1$ for *σ* = 0.1 and 0.2, respectively. For a two photon state, we detect nonclassicality using $0.394 < \eta  < 1$ and $0.443 < \eta  < 1$ for *σ* = 0.1 and 0.2, respectively. Note that the value of *σ* around 0.1 or 0.2 is practically accessible in typical homodyne detection^49^.

## Discussion

In this paper, we have proposed entropic nonclassicality criteria that look into the entropies of a quadrature amplitude before and after a loss channel. Our criteria can be readily tested using homodyne detection with a beam splitter. We have also shown the hierarchical relation among various nonclassicality tests, that is, our entropic tests are strictly stronger than the usual variance-squeezing test. We have illustrated the usefulness of our criteria with non-Gaussian states that do not possess quadrature squeezing but can be detected by our approach.

Furthermore, our approach has been extended to detect quantum non-Gaussianity in conjunction with phase randomization and to detect useful resource states demonstrating quantum steering. In future, we hope our approach here could be further developed to identify a full power of entropic analysis that can be a useful tool to investigate nonclassicality of CV states in general. For instance, our approach can be generalized to adopt Renyi entropies beyond Shannon entropy. It will also be interesting to examine how these entropic criteria can be useful for critical assessment of quantum tasks using continuous variables in relation to QNG^62^.

## Methods

### Integral form of the log sum inequality

The log sum inequality is given by

26 $$\sum _{i}\,{a}_{i}\,\log \,\frac{{a}_{i}}{{b}_{i}}\ge a\,\log \,\frac{a}{b},$$

with $a={\sum }_{i}\,{a}_{i}$ and $b={\sum }_{i}\,{b}_{i}$^23^. We may similarly construct the integral form of the log sum inequality as

27 $${\int }_{\lambda }\,d\lambda p(\lambda )a(\lambda )\,\log \,\frac{a(\lambda )}{b(\lambda )}\ge \overline{a}\,\log \,\frac{\overline{a}}{\overline{b}},$$

where $\overline{o}={\int }_{\lambda }\,d\lambda p(\lambda )o(\lambda )$ with $o\in \{a,b\}$. Using the convexity of $f(x)=x\,\log \,x$ and Jensen’s inequality, we have

28 $$\begin{array}{rcl}{\int }_{\lambda }\,d\lambda p(\lambda )a(\lambda )\,\log \,\frac{a(\lambda )}{b(\lambda )} & = & \overline{b}\,{\int }_{\lambda }\,d\lambda \frac{p(\lambda )b(\lambda )}{\overline{b}}[\frac{a(\lambda )}{b(\lambda )}\,\log \,\frac{a(\lambda )}{b(\lambda )}]\\  & \ge  & \overline{b}[{\int }_{\lambda }\,d\lambda \frac{p(\lambda )b(\lambda )}{\overline{b}}\frac{a(\lambda )}{b(\lambda )}]\\  &  & \times \,\log \,[{\int }_{\lambda }\,d\lambda \frac{p(\lambda )b(\lambda )}{\overline{b}}\frac{a(\lambda )}{b(\lambda )}]\\  & = & \overline{a}\,\log \,\frac{\overline{a}}{\overline{b}}.\end{array}$$

### Proof of Eq. (3)

Using the above integral form of log-sum inequality, we now show that ${\mathcal F} [\rho ]\,\equiv \,{H}_{\rho }(Q)\,-\,{H}_{ {\mathcal L} [\rho ]}(Q)$ defined in Eq. (3) is concave with respect to a non-negative mixture of quantum states, i.e.

29 $$ {\mathcal F} [\rho ]\ge {\int }_{\lambda }\,d\lambda p(\lambda ) {\mathcal F} [{\rho }_{\lambda }],$$

where $\rho ={\int }_{\lambda }\,d\lambda p(\lambda ){\rho }_{\lambda }$ and $p(\lambda )$ is a probability distribution of states ${\rho }_{\lambda }$. Using the fact that the differential entropy of a joint probability distribution is invariant under an orthogonal transformation, we have

30 $$\begin{array}{lcl}{H}_{\rho }(Q)+{H}_{|0\rangle \langle 0|}(Q) & = & -\,{\int }_{-\infty }^{\infty }\,dq{M}_{\rho }(q)\,\log \,{M}_{\rho }(q)-{\int }_{-\infty }^{\infty }\,dq^{\prime} {M}_{|0\rangle \langle 0|}(q^{\prime} )\,\log \,{M}_{|0\rangle \langle 0|}(q^{\prime} )\\  & = & -\,{\int }_{-\infty }^{\infty }\,dq\,{\int }_{-\infty }^{\infty }\,dq^{\prime} {M}_{\rho }(q){M}_{|0\rangle \langle 0|}(q^{\prime} )\,\log \,\{{M}_{\rho }(q){M}_{|0\rangle \langle 0|}(q^{\prime} )\}\\  & = & -\,{\int }_{-\infty }^{\infty }\,dq\,{\int }_{-\infty }^{\infty }\,dq^{\prime} {M}_{\rho }(tq+rq^{\prime} ){M}_{|0\rangle \langle 0|}(\,-\,rq+tq^{\prime} )\\  &  & \times \,\log \,\{{M}_{\rho }(tq+rq^{\prime} ){M}_{|0\rangle \langle 0|}(\,-\,rq+tq^{\prime} )\},\end{array}$$

which can yield

31 $$\begin{array}{rcl} {\mathcal F} [\rho ]+{H}_{|0\rangle \langle 0|}(Q) & = & -\,{\int }_{-\infty }^{\infty }\,dq\,{\int }_{-\infty }^{\infty }\,dq^{\prime} {M}_{\rho }(tq+rq^{\prime} ){M}_{|0\rangle \langle 0|}(\,-\,rq+tq^{\prime} )\\  &  & \times \,\log \,\frac{{M}_{\rho }(tq+rq^{\prime} ){M}_{|0\rangle \langle 0|}(\,-\,rq+tq^{\prime} )}{{M}_{ {\mathcal L} [\rho ]}(q)},\end{array}$$

by using ${M}_{ {\mathcal L} [\rho ]}(q)={\int }_{-\infty }^{\infty }\,dq^{\prime} {M}_{\rho }(tq+rq^{\prime} ){M}_{|0\rangle \langle 0|}(\,-\,rq+tq^{\prime} )$. Employing Eqs. (27) and (31), we obtain

32 $$\begin{array}{lcl} {\mathcal F} [{\int }_{\lambda }\,d\lambda p(\lambda ){\rho }_{\lambda }]+{H}_{|0\rangle \langle 0|}(Q) & = & -\,{\int }_{-\infty }^{\infty }\,dq\,{\int }_{-\infty }^{\infty }\,dq^{\prime} \,{\int }_{\lambda }\,d\lambda p(\lambda ){M}_{{\rho }_{\lambda }}(tq+rq^{\prime} ){M}_{|0\rangle \langle 0|}(\,-\,rq+tq^{\prime} )\\  &  & \times \,\log \,\frac{{\int }_{\lambda }\,d\lambda p(\lambda ){M}_{{\rho }_{\lambda }}(tq+rq^{\prime} ){M}_{|0\rangle \langle 0|}(\,-\,rq+tq^{\prime} )}{{\int }_{\lambda }\,d\lambda p(\lambda ){M}_{ {\mathcal L} [{\rho }_{\lambda }]}(q)}\\  & \ge  & -\,{\int }_{-\infty }^{\infty }\,dq\,{\int }_{-\infty }^{\infty }\,dq^{\prime} \,{\int }_{\lambda }\,d\lambda p(\lambda ){M}_{{\rho }_{\lambda }}(tq+rq^{\prime} ){M}_{|0\rangle \langle 0|}(\,-\,rq+tq^{\prime} )\\  &  & \times \,\log \,\frac{{M}_{{\rho }_{\lambda }}(tq+rq^{\prime} ){M}_{|0\rangle \langle 0|}(\,-\,rq+tq^{\prime} )}{{M}_{ {\mathcal L} [{\rho }_{\lambda }]}(q)}\\  & = & {\int }_{\lambda }\,d\lambda p(\lambda )\{ {\mathcal F} [{\rho }_{\lambda }]+{H}_{|0\rangle \langle 0|}(Q)\}\\  & = & {\int }_{\lambda }\,d\lambda p(\lambda ) {\mathcal F} [{\rho }_{\lambda }]+{H}_{|0\rangle \langle 0|}(Q),\end{array}$$

which yields $$ {\mathcal F} [\rho ]\ge {\int }_{\lambda }\,d\lambda p(\lambda ) {\mathcal F} [{\rho }_{\lambda }]$$.

### Entropic criterion under coarse graining

Investigating the relative entropy between ideal and coarse-grained quadrature distributions, i.e., *M* and $\tilde{M}$, we first find that

33 $$\begin{array}{rcl}D(Q\parallel \tilde{Q}) & = & {\int }_{-\infty }^{\infty }\,dqM(q)\,\log \,M(q)-{\int }_{\infty }^{\infty }\,dqM(q)\,\log \,\tilde{M}(q)\\  & = & -H(Q)-\sum _{n}\,[{\int }_{(n-\frac{1}{2})\sigma }^{(n+\frac{1}{2})\sigma }dqM(q)]\,\log \,\tilde{M}(q)\\  & = & -H(Q)-\sum _{n}\,[{\int }_{(n-\frac{1}{2})\sigma }^{(n+\frac{1}{2})\sigma }\,dq\tilde{M}(q)]\,\log \,\tilde{M}(q)\\  & = & -H(Q)-{\int }_{\infty }^{\infty }\,dq\tilde{M}(q)\,\log \,\tilde{M}(q)\\  & = & -H(Q)+H(\tilde{Q}).\end{array}$$

Due to the non-negativity of the relative entropy, it means that coarse-graining increases the differential entropy, i.e. $H(Q)\le H(\tilde{Q})$. In addition, exploiting the joint convexity of the relative entropy for the case of mixture of coherent states, we have

34 $$\begin{array}{rcl}D(\sum _{k}\,{f}_{k}{Q}_{|{\alpha }_{k}\rangle }\parallel \sum _{k}\,{f}_{k}{\tilde{Q}}_{|{\alpha }_{k}\rangle }) & \le  & \sum _{k}\,{f}_{k}D({Q}_{|\alpha {\rangle }_{k}}\parallel {\tilde{Q}}_{|{\alpha }_{k}\rangle })\\  & \le  & \mathop{{\rm{\max }}}\limits_{|\alpha \rangle }\,D({Q}_{|\alpha \rangle }\parallel {\tilde{Q}}_{|\alpha \rangle }),\end{array}$$

where the maximum relative entropy of the original and the coarse-grained distributions can be efficiently obtained over all coherent state *α* for each *σ* by numerical calculation. Now using $H({\tilde{Q}}_{ {\mathcal L} [\rho ]})-H({Q}_{ {\mathcal L} [\rho ]})\le {{\rm{\max }}}_{|\alpha \rangle }\,D({Q}_{|\alpha \rangle }\parallel {\tilde{Q}}_{|\alpha \rangle })$ for the case of classical states (mixture of coherent states) and $H({\tilde{Q}}_{\rho })\ge H({Q}_{\rho })$, we obtain the classiscality condition as

35 $$\begin{array}{rcl}H({\tilde{Q}}_{ {\mathcal L} [\rho ]})-H({\tilde{Q}}_{\rho }) & \le  & H({Q}_{ {\mathcal L} [\rho ]})+\mathop{{\rm{\max }}}\limits_{|\alpha \rangle }\,D({Q}_{|\alpha \rangle }\parallel {\tilde{Q}}_{|\alpha \rangle })-H({Q}_{\rho })\\  & \le  & \mathop{{\rm{\max }}}\limits_{|\alpha \rangle }\,D({Q}_{|\alpha \rangle }\parallel {\tilde{Q}}_{|\alpha \rangle })\equiv {B}_{\sigma }.\end{array}$$

where we have used the classicality condition for the ideal distributions, i.e. $H({Q}_{ {\mathcal L} [\rho ]})\le H({Q}_{\rho })$. In other words, if the output entropy is larger than the input entropy as $H({\tilde{Q}}_{ {\mathcal L} [\rho ]}) > H({\tilde{Q}}_{\rho })+{B}_{\sigma }$, the state is nonclassical. We numerically obtain *B*_*σ*_ = 0.0016639 and 0.0066226 for $\sigma =0.1$ and $\sigma =0.2$, respectively, which are used in main text.
